# RIG-I and dsRNA-Induced IFNβ Activation

**DOI:** 10.1371/journal.pone.0003965

**Published:** 2008-12-30

**Authors:** Stéphane Hausmann, Jean-Baptiste Marq, Caroline Tapparel, Daniel Kolakofsky, Dominique Garcin

**Affiliations:** 1 Department Of Microbiology and Molecular Medicine, University of Geneva Medical School, Geneva, Switzerland; 2 Laboratory of Virology, Division of Infectious Diseases, University of Geneva Hospitals and Faculty of Medicine, Geneva, Switzerland; Instituto Oswaldo Cruz and FIOCRUZ, Brazil

## Abstract

Except for viruses that initiate RNA synthesis with a protein primer (e.g., picornaviruses), most RNA viruses initiate RNA synthesis with an NTP, and at least some of their viral _ppp_RNAs remain unblocked during the infection. Consistent with this, most viruses require RIG-I to mount an innate immune response, whereas picornaviruses require mda-5. We have examined a SeV infection whose ability to induce interferon depends on the generation of capped dsRNA (without free 5′ tri-phosphate ends), and found that this infection as well requires RIG-I and not mda-5. We also provide evidence that RIG-I interacts with poly-I/C *in vivo*, and that heteropolymeric dsRNA and poly-I/C interact directly with RIG-I *in vitro*, but in different ways; i.e., poly-I/C has the unique ability to stimulate the helicase ATPase of RIG-I variants which lack the C-terminal regulatory domain.

## Introduction

Virus infection elicits potent cellular responses that contain virus spread before the adaptive immune system can intervene, and the production of type I interferons (IFNα/β) is central to this process [1], [2]. The sensors involved in coupling recognition of virus infection with the induction of IFNα/β have recently been discovered. These sensors, or pattern recognition receptors (PRRs) that recognize pathogen associated molecular patterns (PAMPs), include RIG-I and mda-5, two cytoplasmic, RNA-binding DExD/H box helicases (for recent reviews, see [3]–[5]. Both proteins contain N-terminal CARD domains, followed by a DECH box helicase. Both proteins also contain a C-terminal domain, which in the case of RIG-I acts as an internal repressor or regulatory domain (RD) that prevents the CARDs from interacting with their downstream signaling adaptor, IPS-1 [6]. The binding of 5′ tri-phosphorylated RNA (_ppp_RNA, which acts as a viral PAMP [7], [8]) to the RD of RIG-I leads to its dimerization, which is thought to stimulate the helicase ATPase and release the CARDs for homotypic interaction with IPS-1 [9], the mitochondrial adaptor of both RIG-I and mda-5. IPS-1 activation then leads to the recruitment of a series of kinases which in turn leads to the activation of IRF-3/7 and NF-kB and the expression of the early IFN genes, such as IFNβ.

When RIG-I was first described in the seminal paper of Yoneyama et al [10], poly-I/C was proposed as its ligand based on RIG-I over-expression. RIG-I deficient mice, however, were then found not to be defective in their type I IFN response to poly-I/C [11], whereas they were unable to mount an innate immune response to most RNA viruses other than picornaviruses like EMCV (e.g., Influenza A virus, VSV, JEV and Sendai virus (SeV) [12]. Mda-5 deficient mice, in contrast, were found to be entirely unable to mount a type I IFN response to poly-I/C and to EMCV infection [12], [13]. The role of these two helicases in the innate immune response to virus infection was thus found to be remarkably specific. Using cell lines derived from these mice, mda-5^−/−^ MEFs were found to activate IFN genes in response to various transfected dsRNAs made from complementary _ppp_RNAs transcribed *in vitro*, whereas these MEFs did not respond to poly-I/C. In contrast, RIG-I^−/−^ MEFs activated IFN genes in response to transfected poly-I/C, but these MEFs did not respond to dsRNAs made from *in vitro* transcripts [12], [13]. Subsequently, ssRNA transcribed *in vitro* was also found to be a ligand for RIG-I, and its ability to induce IFN upon transfection depended on the 5′ triphosphate moiety of the ssRNA [7], [8]. Thus, RIG-I was thought to act as a PRR exclusively for _ppp_RNA (independent of its single- or double-strandedness), and mda-5 for poly-I/C, or more realistically for the RNA elements of picornavirus infection that are mimicked by poly-I/C.

RIG-I and mda-5 are thus thought to recognize different RNA ligands (_ppp_RNA and poly-I/C or dsRNA, respectively) that act as PAMPs, which presumably accounts for the virus-specific response of these helicases. This is consistent with our view of RNA virus replication. Except for picornaviruses (and caliciviruses) that initiate all RNA synthesis with a protein primer; the other RNA viruses initiate all RNA synthesis with an NTP, and at least some of the viral _ppp_RNAs remain unblocked during the infection (e.g., the minus-strands of plus-strand and dsRNA viruses) [14]. Thus, except for picornaviruses (and possibly caliciviruses), cells require RIG-I (and not mda-5) to activate IFNβ in response to other RNA virus infections. In order to test this contention, we have designed a SeV infection that generates dsRNA with capped 5′ ends [15] to examine whether this SeV infection requires mda-5 rather than RIG-I to activate IFNβ. Remarkably, this dsRNA-generating SeV co-infection also requires RIG-I [and not mda-5] to activate IFNβ. This study also provides evidence that RIG-I binds dsRNA devoid of free 5′ tri-phosphate ends, and that poly-I/C is not a simple analog of dsRNA; i.e., poly-I/C has the unique ability to stimulate the helicase ATPase of RIG-I variants which lack the C-terminal regulatory domain.

## Materials and Methods

### Cells, viruses, and antibodies

RIG-I^−/−^ and mda-5^−/−^ MEFs were obtained from H Kato and S Akira, Osaka, Japan [11], [12]. All cells were grown in Dulbecco's modified Eagle's medium supplemented with 10% fetal calf serum.

SeV-GFP(+), which expresses green fluorescent protein (GFP) from a transgene between the M and F genes, and SeV-GFP(−), which expresses antisense GFP mRNA from a similarly located transgene, were prepared as previously described [16]. DI-H4 stocks were described previously [17].

Primary antibodies used included anti-Flag MAb (F1804; Sigma), rabbit anti-mda-5 and mouse anti-Rig-I (J. Tschopp, Lausanne, Switzerland). Rabbit anti-RIG-I which reacts with both the human and murine helicases was provided by S. Akira (Osaka, Japan).

### Plasmids, transient transfections, infections, inductions, and luciferase assay

Flag-tagged RIG-I, and mda-5 were obtained from Klaus Conzelmann (Munich) and Jurg Tshopp (Lausanne). Mda5-ΔCARD was obtained from S Goodbourn (London). N-terminal deletion mutants of RIG-I (residues 242–925) were constructed by PCR amplification with mutagenic sense primers that introduced a Kpn I site and a met codon in lieu of phe241. C-terminal deletion mutants were constructed with antisense primers that introduced a stop codon and a *Kpn* site in lieu of Pro 797. The PCR products were digested with *kpn* and then inserted into pEF-BOS (kindly provided by J. Tschopp). The inserts of the resulting pEF-BOS Rig-I plasmids were confirmed by sequencing.

pβ-IFN-fl-lucter, which contains the firefly luciferase gene under the control of the human IFN-β promoter, was described previously [18]. pTK-rl-lucter, used as a transfection standard, contains the herpes simplex virus TK promoter region upstream of the *Renilla* luciferase gene (Promega).

#### Transfections

100,000 cells were plated into six-well plates 20 h before transfection with 1.5 µg of pβ-IFN-fl-lucter; 0.5 µg of pTK-rl-lucter; 1 µg of plasmids expressing RIG-I and MDA-5; 1.5 µg of plasmids expressing RIG-ΔCARD, Mda-ΔCARD (as indicated); and TransIT-LT1 transfection reagent (Mirus). At 24 h posttransfection, the cells were (or were not) infected with various SeV stocks or transfected with 5 µg of poly(I-C) using TransIT-LT1 transfection reagent. Twenty hours later, cells were harvested and assayed for firefly and *Renilla* luciferase activity (dual-luciferase reporter assay system; Promega). Relative expression levels were calculated by dividing the firefly luciferase values by those of *Renilla* luciferase.

#### Immunoblotting

Cytoplasmic extracts were prepared using 0.5% NP-40 buffer. Equal amounts of total proteins were separated by SDS-PAGE and transferred onto Immobilon-P membranes by semi-dry transfer. The secondary antibodies used were alkaline phosphatase-conjugated goat anti-rabbit (or mouse) immunoglobulin G (Bio-Rad). The immobilized proteins were detected by light-enhanced chemiluminescence (Pierce) and analyzed in a Bio-Rad light detector using Quantity One software.

#### Recombinant RIG-I cloning and expression

The open reading frame of human RIG-I was amplified by PCR using primers designed to introduce a HindIII site upstream of the start codon and a XhoI site downstream. The PCR products were digested and then inserted between the same sites of pET28-His_10_Smt3, to fuse the RIG-I proteins in-frame with an amino-terminal His_10_Smt3 domain. RIG-I (1–796) was constructed with an antisense primer that introduced a stop codon in place of pro797 and a XhoI site. RIG-I (242–796) was constructed with sense primer that introduced a HindIIII site upstream of lys241and the antisense primer used above. The K270A mutation was introduced by a PCR-based two-stage overlap extension method. The plasmid inserts were sequenced completely to ensure that unwanted mutations during amplification and cloning had not occurred.

The pET28-His_10_Smt3-Tgs1 plasmids were transformed into *E. coli* BL21. Cultures (500 ml) derived from single transformants were grown at 37°C in LB medium containing 50 µg/ml kanamycin until the *A*_600_ reached 0.6. The cultures were adjusted to 0.2 mM IPTG and 2% ethanol and incubation was continued for 20 h at 17°. Cells were harvested by centrifugation and stored at −80°C. All subsequent procedures were performed at 4°. Thawed bacteria were resuspended in 25 ml of buffer A (50 mM Tris-HCl, pH 8.0, 200 mM NaCl, 10% glycerol) and one tablet of protease inhibitor cocktail (Roche) and lysozyme (100 µg/ml) were added. After incubation for 30 min, imidazole was added to a final concentration of 5 mM and the lysate was sonicated to reduce viscosity. Insoluble material was removed by centrifugation. The soluble extracts were mixed for 30 min with 1.6 ml of Ni^2+^-NTA-agarose (Qiagen) that had been equilibrated with buffer A containing 5 mM imidazole. The resins were recovered by centrifugation, resuspended in buffer A containing 5 mM imidazole, and poured into columns. The columns were washed with 8 ml of 10 and 20 mM imidazole in buffer A and then eluted step-wise with 2.5 ml aliquots of buffer A containing 50, 100, 250, and 500 mM imidazole. The elution profiles were monitored by SDS-PAGE. The 250 mM imidazole eluates containing the recombinant RIG-I polypeptides were dialyzed against 50 mM Tris-HCl, pH 8.0, 200 mM NaCl, 2 mM DTT, 1 mM EDTA, 10% glycerol and then stored at −80°C. The protein concentration was determined using the Bio-Rad dye binding method with BSA as the standard.

### qRT/PCR of endogenous IFNβ mRNA

RNA was extracted from cell lysates with TRIzol reagent (Invitrogen) and reverse transcribed with random hexamers (Roche) and Reverse Transcriptase Superscript II (Invitrogen) according to manufacturer's instructions. The cDNA was then amplified using a TaqMan® 7500 (Applied Biosystems) thermocycler. Results were analysed using the SDS 1.4 programme (Applied Biosystems). 18S rRNA primers and probe sequences used were as described previously [15]. Murine beta IFN primers and probes used were as described in [19].

#### In vitro synthesis of RNA, purification, and transfection

DNA for T7 RNA polymerase synthesis of model RNA1 was prepared by PCR using the following partially complementary primers: 5′-TAATACGACTCACTATAgggACACACCACAACCAACCCACAAC-3′ (forward) (start sites are in lowercase type) and 5′-GAAAGAAAGGTGTGGTGTTGGTGTGGTTGTTGTGGGTTGGTTGTGG-3′ (reverse). Transcription was performed on 100 pmol of purified PCR product using T7 MEGAshortcript from Ambion, according to the manufacturer's instructions. Biotinylated RNA1 was synthesized using equal amounts of 5′ biotin-UTP and UTP. The T7 transcripts were purified on NucAway Spin columns from Ambion (to remove unincorporated nucleotides).

For RNA transfection, RNA was transfected into MEFss using TransMessenger transfection reagent (QIAGEN).

The various homo-polymers were from Sigma. 5′ OH chemically synthesized Tr41 (the first 41 nt of the SeV trailer RNA) and its complement, as well Tr41 with a 3′ C_12_ extension (53′mer) were purchased from MicroSynth. TransIT-LT1 transfection reagent was from Mirus.

### _ppp_ssRNA beads

Streptavidin agarose beads (Fluka 85881) were pre-equilibrated with Blocking buffer, i.e., Base buffer (20 mM Hepes pH 7.9, 2 mM EDTA, 15% glycerol, 0.05% NP40, 50 mM NaCl, 500 unit/ml RNasin (Promega N2515), 0.02 mg/ml tRNA (Roche 10109495001), 1% protease inhibitor cocktail (Sigma P8340) and 2 mM DTT) plus 100 mM NaCl, another 100 unit/ml RNasin, 0.1 mg/ml glycogen and 2.5 mg/ml BSA, for 2 hours at 4°C. Biotinylated RNA was bound to the beads in Base buffer for 2 hours at 4°C. Beads to which 1.5 ug of RNA were added were used for each assay.

## Results

### SeV Infections of RIG-I^−/−^ and mda-5^−/−^ MEFs

Unnatural SeV infection is commonly used to induce IFN, as wt SeV infection induces IFN very poorly [17]. This is because the wt SeV genome expresses C and V proteins which counteract the innate immune response in several ways, most notably by their ability to inhibit IFN activation by transfected _ppp_RNA and poly-I/C [15]. The extent to which IFNβ is activated during SeV infection presumably depends both on the level of the RNA PAMPs produced and that of the viral products that counteract the innate immune response. For mononegaviruses whose genome and antigenome RNAs are tightly covered with N protein during their synthesis (and thus are unlikely to act as PAMPs), small promoter-proximal (leader and trailer) _ppp_ RNAs are made independent of assembly with N [15], [20], [21]. These promoter-proximal _ppp_ RNAs are essential for the control of genome replication. Viral dsRNA could also be generated by the annealing of trailer _ppp_RNA and L mRNA read-thru transcripts. During wt SeV infection of non-immune cells, the activity of both PAMPs is presumably neutralized by the viral C proteins [15]. Akira and coworkers have previously used SeV-C^minus^ infection to induce IFN [12]. The SeV which is more commonly used to induce IFN (“Cantell strain”) is in fact a mixed virus stock composed mostly of copyback defective-interfering (DI) genomes, and this DI infection both under-produces the C and V proteins and overproduces trailer _ppp_RNA. In addition, because some of the copyback DI genomes are exceedingly small (546 nt vs 15,264 for wt), some of these genomes may also be made without being assembled with N [17]. If so, these unassembled DI genomes would self-anneal to form _ppp_dsRNA panhandle structures because their ends are self-complementary [22]. Thus, both _ppp_ssRNA and _ppp_dsRNA (i.e., in which one strand contains a 5′ tri-phosphate end) are thought to induce IFNβ activation during SeV-C^minus^ and DI infections.

More recently, we have also used quasi-wt SeV co-infections that express GFP mRNA and anti-GFP mRNA (from separate genomes) to activate IFNβ [15]. The ability of this co-infection to induce IFN depended on the presence of both complementary GFP mRNAs in the cytoplasm, and RFP mRNA expression could not substitute for one of the GFP mRNAs. IFNβ activation induced by the GFP+/− co-infection thus presumably results from the generation of GFP dsRNA in which both 5′ ends are capped. Moreover, this IFNβ activation also appeared to depend on RIG-I, as a dominant-negative form of RIG-I (RIG-I-ΔCARD) inhibited this response. However, the precise manner in which RIG-I-ΔCARD acts as a dominant-negative inhibitor of RIG-I is not known, and this mutant helicase may be acting non-specifically when over-expressed.

**Figure 1:**
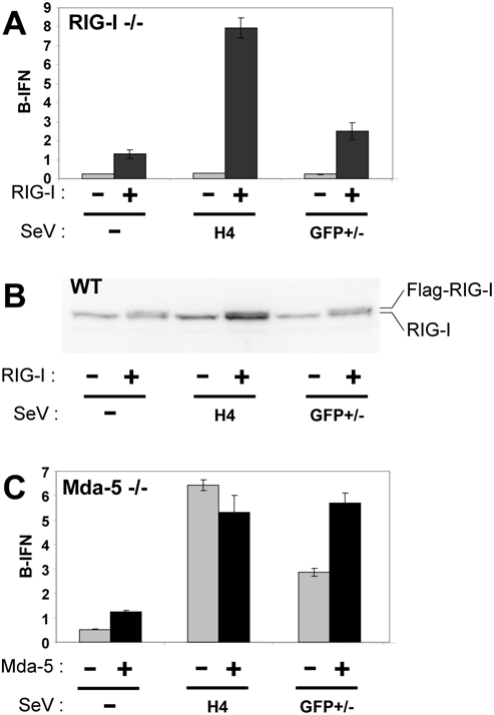
SeV infection of RIG-I^−/−^ and mda-5^−/−^ MEFs. RIG-I^−/−^ (panel A) and mda-5^−/−^ MEFs (panel C) were transfected with pIFNβ-(ff)luciferase and pTK-(ren)luciferase, plus and minus pEBS-Flag-RIG-I and pEBS-Flag-mda-5, respectively. Parallel cultures were then mock-infected, or infected with either 20 pfu/cell of SeV-GFP+ and SeV-GFP− (GFP+/−), or an equivalent amount of SeV-DI-H4 (H4) in duplicate at 24 h post-transfection. Cell extracts were prepared at 20 h.p.i. and their luciferase activities were determined. This experiment was repeated twice with similar results. Wild-type MEFs were also transfected with pEBS-Flag-RIG-I and pGFP. At 24 hpt, some of the cells were analyzed by FACS to determine the fraction expressing GFP. Cell extracts were prepared at this time and Western blotted with antisera that recognize both the human and murine forms of RIG-I (panel B).

To further investigate the helicase requirement for the GFP+/− infection, RIG-I^−/−^ and mda-5^−/−^ MEFs were transfected with plasmids expressing luciferase under the control of the IFNβ promoter (and control plasmids), and then infected with SeV DI-H4 or GFP+/− stocks. Cells extracts were prepared at 20 hpi and their luciferase activities were determined. As shown in fig 1A, neither infection of RIG-I^−/−^ MEFs activated the IFNβ promoter above background levels. When RIG-I was re-expressed in these cells by transfection (along with the reporter plasmids), this restored the ability of both infections to activate IFNβ. In contrast, the DI-H4 and GFP+/− infections of mda-5^−/−^ MEFs activated the IFNβ promoter in both cases. The re-expression of mda-5 in these cells roughly doubled their response to the GFP+/− infection, but did not stimulate their response to the DI-H4 infection (fig 1C). Thus, the presence of RIG-I is essential for IFNβ activation during GFP+/− infection that generate capped-dsRNA, as well as during DI-H4 infections. In contrast, the presence of mda-5 is not essential, but can stimulate IFNβ activation in response to capped dsRNA in mda-5−/− cells.

To determine the levels of RIG-I generated by transfection relative to those of the endogenous helicase in MEFs, wt MEFs were also transfected with (human) p-Flag-RIG-I (and pGFP as a transfection indicator) under identical conditions as in panels A and C. Cell extracts were then Western blotted with an antibody that reacts with both human and murine RIG-I. This estimation of RIG-I levels is also more informative in wt MEFs because they are efficiently transfected (ca 50%), in contrast to the helicase-deficient MEFs that are poorly transfected (<5%). As shown in panel B, the level of flag-RIG-I expressed by transfection (the upper band of the doublet) is estimated to be 50–100% as strong as the endogenous band. Given that 48% of these wt MEFs were transfected (as indicated by GFP expression), Flag-RIG-I is expressed under these condition at levels that are 1 to 2 times those of the endogenous helicase (assuming that our anti-RIG-I reacts equally with both forms of the helicase).

### Interaction of RIG-I with poly-I/C *in vivo*

We would of course like to confirm that RIG-I also senses dsRNA without 5′ tri-phosphate ends, by examining whether capped GFP dsRNA (from SeV infected cells) activates IFNβ upon transfection. SeV infected cells, however, will also contain viral leader and trailer _ppp_ssRNAs, and _ppp_dsRNAs (made from trailer RNA and L mRNA read-through transcripts), and determining the level of purity of the GFP dsRNA is problematic. We have also tried to prepare such capped dsRNA by capping GFP transcripts (made *in vitro*) with the vaccinia virus guanylyl transferase, but we were unable to cap more than 70% of each strand. Natural dsRNA that can be obtained in pure form, like reovirus RNA, contains a free 5′ tri-phosphate (minus-strand) end. We therefore turned to poly-I/C that contains 5′ di-phosphate ends, as model RNAs containing these 5′ ends were found not to activate RIG-I [8].

We examined the possible interaction of poly-I/C and RIG-I in MEFs using helicases that lack their N-terminal CARDs, which appear to act as dominant-negative inhibitors of the helicases. For example, over-expression of the tandem CARDs of RIG-I alone induce IFN independently of the presence of viral RNA, suggesting that the CARDs mediate IPS-1 activation and downstream signaling [10]. Mutation of the RIG-I ATP binding site abolishes RIG-I activity, suggesting that ATP and RNA-dependent conformational changes are essential for sensing viral RNA [10]. Finally, over-expression of the RIG-I RD alone inhibits RIG-I signaling in response to SeV DI infection, by apparently interfering with the oligomerization of wt RIG-I [6]. RIG-I-ΔCARD could then act as a dominant-negative inhibitor of RIG-I because of its ability to bind viral RNAs and oligomerize with wt RIG-I, but this mixed oligomer would not activate IPS-1. Less is known about the manner in which mda-5 signals to IFNβ.

**Figure 2:**
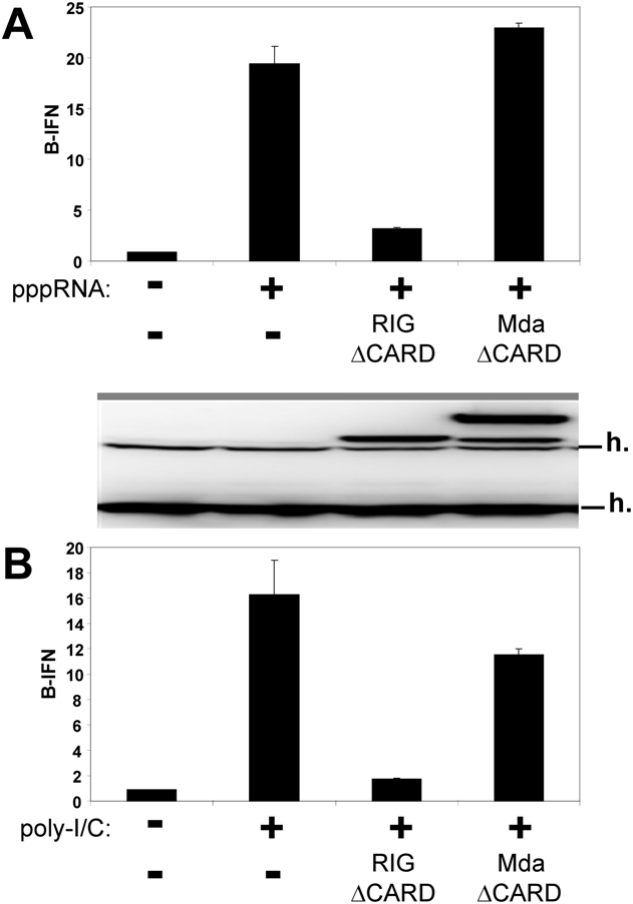
The effect of CARD-less forms of RIG-I and mda-5 on _ppp_RNA and poly-I/C induced IFNβ activation. Parallel cultures of wt MEFs were transfected with pIFNβ-(ff)luciferase and pTK-(ren)luciferase, plus and minus either pEBS-RIG-I-ΔCARD (residues 242–925) or pEBS-mda-5-ΔCARD (residues 197–1025). 24 h later, these cells were transfected in duplicate with either 3 ug _ppp_(ss)RNA1 (panel A) or 5 ug of poly-I/C (panel B). Cell extracts were prepared after a further 20 h, and their luciferase activities were determined. Equal amounts of cell extracts (total protein) were Western blotted with anti-flag and anti-mda-5. Cross-reacting host bands (h) serve as an internal loading control.

**Figure 3:**
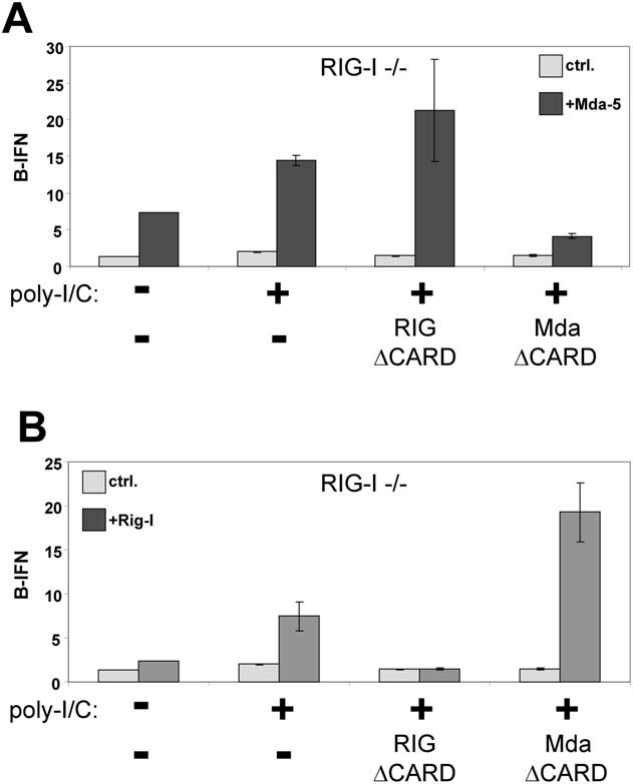
Effect of CARD-less helicases on poly-I/C induced IFNβ activation. Parallel cultures of RIG-I^−/−^ MEFs were transfected with pIFNβ-(ff)luciferase and pTK-(ren)luciferase. Some of the cultures were (or were not) transfected with plasmids expressing mda-5 (panel A; +mda-5 and ctrl) or RIG-I (panel B; +RIG-I and ctrl), along with plasmids expressing RIG-I-ΔCARD or mda-5-ΔCARD as indicated at the bottom of each panel. 24 h later, the cultures were transfected in duplicate with 5 ug of poly-I/C, as imdicated. Cell extracts were prepared after a further 20 h, and their luciferase activities were determined.

When IFNβ activation in MEFs in response to transfected _ppp_ssRNA or poly-I/C is compared, this activation is largely inhibited by the co-expression of RIG-I-ΔCARD in both cases (fig 2). In contrast, the co-expression of mda-5-ΔCARD has no effect on the activation induced by _ppp_ssRNA, and a mimimal effect on that induced by poly-I/C. This does not appear to be because mda-5-ΔCARD is inactive, or because RIG-I-ΔCARD is acting non-specifically. When the CARD-less helicases are expressed in RIG-I^−/−^ MEFs in which mda-5 is (or is not) expressed by transfection, mda-5-ΔCARD clearly inhibits the poly-I/C induced activation due to the (over-)expressed mda-5, whereas RIG-I-ΔCARD does not at all inhibit this activation that is exclusively due to the presence of mda-5 (fig 3A). In contrast, when the CARD-less helicases are expressed in RIG-I^−/−^ MEFs in which RIG-I is (or is not) expressed, mda-5-ΔCARD now does not inhibit (but rather stimulates) the poly-I/C induced activation due to the (over-)expressed RIG-I, whereas RIG-I-ΔCARD clearly inhibits this activation in the presence of both helicases (fig 3B). Thus, mda-5-ΔCARD does indeed act as an inhibitor of poly-I/C induced IFNβ activation, but only when this activation is due exclusively to mda-5. When both helicases are present, it is RIG-I-ΔCARD (and not mda-5-ΔCARD) that inhibits poly-I/C induced IFNβ activation.

**Figure 4:**
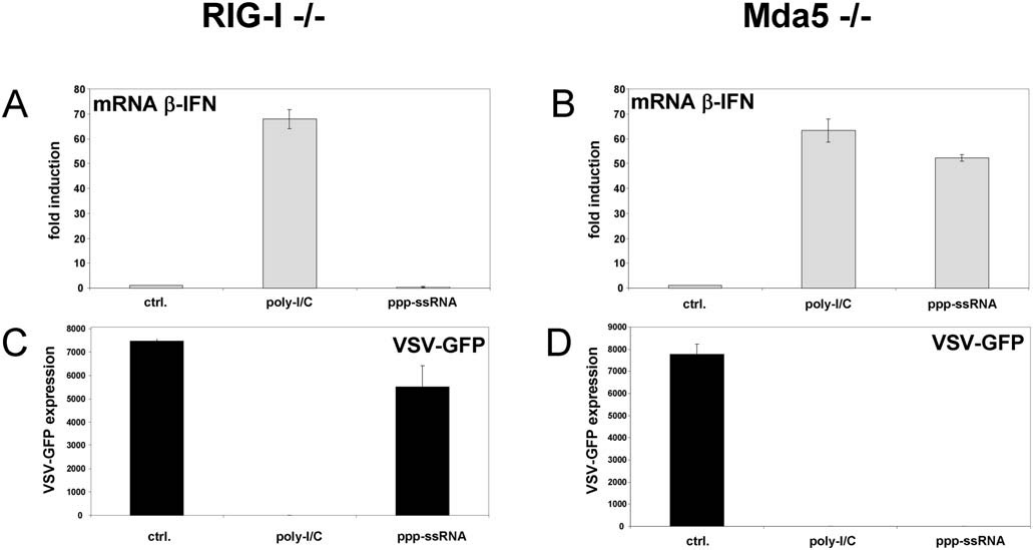
Poly-I/C and _ppp_RNA induced activation of endogenous IFNβ in helicase-deficient MEFs. RIG-I^−/−^ (panel A) and mda-5^−/−^ MEFs (panel B) were transfected with either 5 ug of poly-I/C, 3 ug of _ppp_RNA1 (55 nt) or empty transfection mix (ctrl) as indicated. Cell extracts were prepared at 20 hpt, and the relative levels of endogenous IFNβ mRNA were determined by qRT/PCR (Methods). The cell supernatants of the transfected cells were also harvested at this time, and used to pretreat wt MEFs for 12 h before infection with VSV-GFP. The VSV infected cells were harvested at 12 hpi and analyzed by FACS for GFP expression (panels C and D).

Although the helicase-deficient MEFs transfect poorly with plasmid DNA, they appear to be more efficiently transfected with either relatively small poly-I/C (400 bp on average) or _ppp_RNA (55 nt)(fig 4). When RIG-I^−/−^ MEFs are transfected with poly-I/C or _ppp_RNA, the level of endogenous IFNβ mRNA increases only in response to poly-I/C as expected (fig 4A), as mda-5 does not respond to _ppp_RNA [12]. The increased IFNβ mRNA apparently leads to the secretion of IFN, as pretreatment of wt MEFs with the supernatants from the above experiment efficiently prevented the growth of VSV-GFP in these cells only when poly-I/C had been transfected (fig 4C). In contrast, when mda-5^−/−^ MEFs are transfected with poly-I/C or _ppp_RNA, the level of endogenous IFNβ mRNA increases in response to poly-I/C as well as to _ppp_RNA (fig 4B), and the supernatants from both these transfections have the capacity to inhibit VSV-GFP replication when used to pretreat other MEFs (fig 4D). These results further indicate that RIG-I responds to poly-I/C as well as to _ppp_RNA.

**Figure 5:**
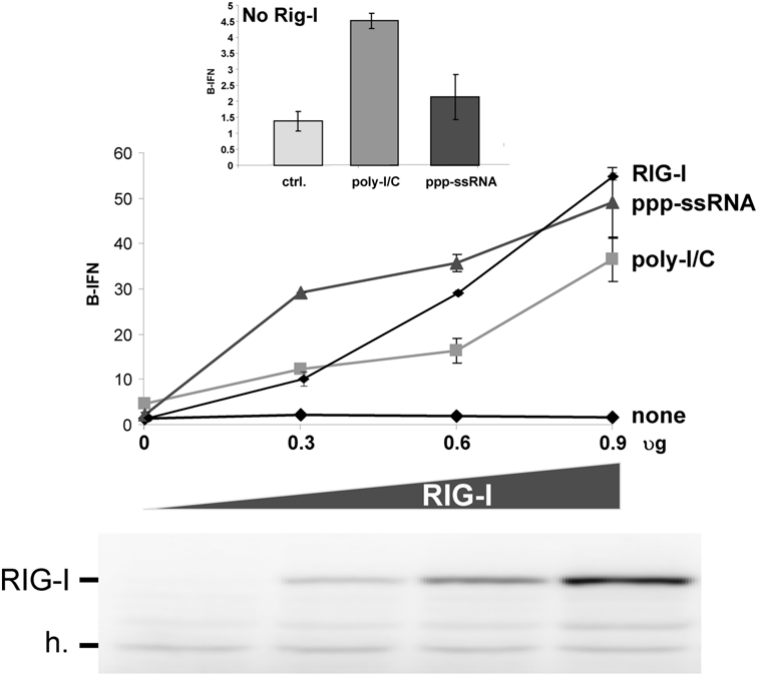
Effect of increasing RIG-I expression on RNA-induced IFNβ activation. Parallel cultures of RIG-I^−/−^ MEFs were transfected with pIFNβ-(ff)luciferase and pTK-(ren)luciferase. Some of the cultures were transfected with increasing amounts of plasmids expressing RIG-I. 24 h later, the cultures were transfected in duplicate (or not (none)) with either 3 ug _ppp_(ss)RNA1 or 5 ug of poly-I/C. Cell extracts were prepared after a further 20 h, and their luciferase activities were determined. Equal amounts of cell extracts (total protein) were Western blotted with anti-RIG-I. Cross-reacting host bands (h) serve as an internal loading control. The intensities of the RIG-I bands were determined by densitometry and their relative values (RIG-I levels) are plotted above. The insert above shows a blow-up of the IFNβ activation in the absence of any RIG-I.

We also examined whether the level of IFNβ activation was proportional to that of RIG-I expression in RIG-I^−/−^ MEFs. Increasing amounts of RIG-I were expressed in these cells, which were then subsequently transfected with either _ppp_ssRNA or poly-I/C. Expression of increasing amounts of RIG-I had little or no effect on IFNβ activation in the absence of transfected RNA (none, fig 5). In the presence of transfected RNA, the level of IFNβ activation was indeed proportional to that of RIG-I expression for both poly-I/C and _ppp_ssRNA, and poly-I/C was, remarkably, half as efficient as _ppp_ssRNA. Although it is possible that the combined effect of poly-I/C and increasing RIG-I levels act indirectly to increase IFNβ activation (e.g., by increasing mda-5 levels), the fact that this increase in IFNβ activation depends on the presence of both poly-I/C and increased RIG-I suggests that RIG-I can interacts with poly-I/C *in vivo*.

### Interaction of poly-I/C and RIG-I *in vitro*

**Figure 6:**
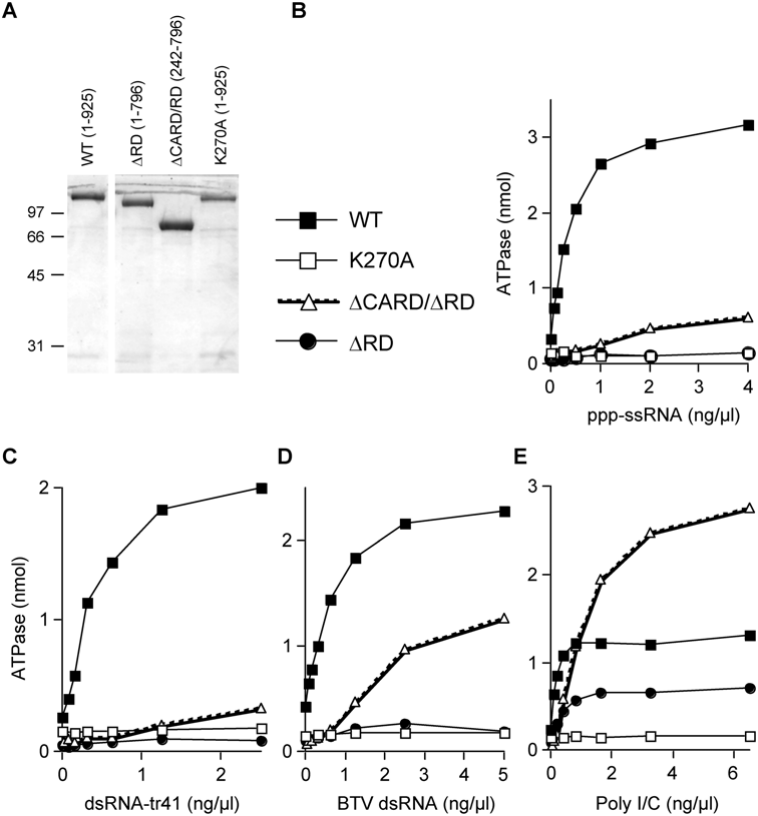
ATPase activity of RIG-I variants. (*A*) Samples (5 µg) of the dialyzed nickel-agarose fractions of wt Rig-I (1–925), Rig-I-ΔRD (1–796), Rig-I-ΔCARD/ΔRD (242–796), and Rig-I-K270A were electrophoresed on a 10% SDS gel and visualized by Coomassie blue staining. The positions of marker proteins are indicated on the left. (B to E) Effect of various RNAs on ATPase activity. Reaction mixtures (15 µl) containing 50 mM Tris–acetate pH 6.0, 5 mM DTT, 1.5 mM MgCl_2_, 500 µM [γ-^32^P]ATP, increasing amounts of either ppp-ssRNA (panel B), dsRNA-tr41 (panel C), BTV dsRNA (panel D), or poly-I/C (panel E) as indicated, and 200 ng of recombinant proteins were incubated for 15 min at 37°. The reactions were quenched by adding 3.8 µl of 5 M formic acid. Aliquots (3 µl) of the mixtures were applied to a polyethyleneimine–cellulose thin-layer chromatography (TLC) plates and developed with 1 M formic acid and 0.5 M LiCl. ^32^PO_4_ release was quantified with a phosphorimager. The results are plotted as a function of RNA concentration.

DExH/D box helicases share 7 conserved sequence motifs that mediate ATP and nucleic acid binding [23]. Nucleic acid binding stimulates the helicase ATPase and results in a conformational power stroke [24]. ATP binding is essential for RIG-I signaling, but the mechanistic role of the ATPase activity in RIG-I signaling to IFNβ is unclear. RIG-I is required for non-immune cells to mount an IFN response to SeV-C^minus^ and DI-H4 infections, which presumably express different levels of _ppp_ssRNA and _ppp_dsRNA. RIG-I is also required for non-immune cells to mount an IFN response to capped dsRNA (i.e., without any free 5′ tri-phosphate ends). Consistent with these results, the RIG-I ATPase is stimulated not only by _ppp_ssRNA, but also by several dsRNAs, including those made by annealing chemically synthesized complementary RNAs (dsRNA-tr41, containing two 5′-OH ends)(fig 6C), bluetongue (reo)virus dsRNA (BTV RNA; 10 segments ranging from ca. 400 to 4000 bp, which contain one capped and one 5′-ppp end) (fig 6D), and poly-I/C, poly-G/C and poly-A/U (all originally containing 5′-pp ends)(fig 6E and data not shown). In contrast, the RIG-I ATPase is not stimulated by chemically synthesized _OH_ssRNA (data not shown) [9]. Cui et al have reported that_ ppp_ssRNA is much more efficient in stimulating wt RIG-I (1–925) ATPase than _OH_dsRNA. This difference is less pronounced, but clear in fig 6B and C.

**Figure 7:**
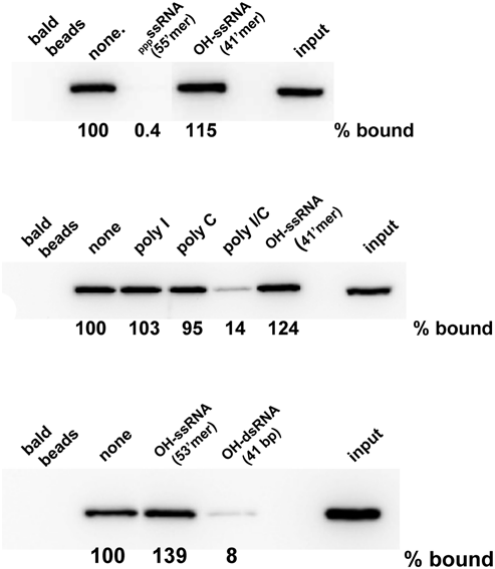
Competition of various RNAs for RIG-I binding to pppRNA beads. Streptavidin beads to which 1.5 ug of biotinylated _ppp_RNA1 had been added (or not; bald beads) were incubated with 1 ug of purified RIG-I (Methods) and 5 ug of various competitor RNAs as indicated. After 2 h at 4°, the beads were washed 3 times with base buffer, SDS protein sample buffer was added, and the beads were Western blotted with anti-RIG-I. The amount of RIG-I remaining on the beads (% bound) relative to that in the absence of competitor is given below each panel. The right-hand lanes “input” show 1/8 of the RIG-I used for the RNA binding that were directed Western blotted (i.e., 1/8 of the input).

Two groups have recently reported that the RD of RIG-I alone binds _ppp_ssRNA, in a shallow, positively-charged groove [9], [25]. These two studies were largely in agreement, but differed in that the RD in one case also bound relatively short _OH_dsRNA (25 bp)[25], whereas that of Cui et al did not interact with_ OH_dsRNA (50 bp) even though the ATPase of a RIG-I variant lacking the CARDs was clearly stimulated by _OH_dsRNA [9]. We have used streptavidin beads containing biotinylated_ ppp_ssRNA to study RNA/RIG-I interaction. As shown in fig 7, RIG-I binds to_ ppp_ssRNA beads (lanes “none” (no competitor) vs bald beads). As expected, this binding can be efficiently out-competed with an excess of unmodified _ppp_ssRNA (55′mer, top panel), but the same amount of _OH_ssRNA (41′mer, top and middle panels), poly-I or poly-C (middle panel) had no effect. More importantly, RIG-I binding to_ ppp_ssRNA can be efficiently out-competed with relatively short _OH_dsRNA (41 bp, bottom panel) under conditions where an equal amount of _OH_ssRNA (41′mer or 53′mer (bottom panel)) has no effect. Poly-I/C also competed with the binding of RIG-I to _ppp_ssRNA (middle panel). Thus, the binding of short _OH_dsRNA or poly-I/C and _ppp_ssRNA to RIG-I appear to be mutually exclusive, either because their binding sites on RD overlap [25], or because _OH_dsRNA binds to the helicase core in an RD-dependent manner. This latter possibility is suggested by the finding that the ATPase of a RIG-I variant lacking RD (residues 1–793) could not be stimulated by either _ppp_ssRNA or _OH_dsRNA [9].

We have repeated and extended these ATPase studies, using RIG-I-ΔRD (in this case residues 1–796) and RIG-I-ΔCARD/ΔRD (242–796), as well as full-length (wt) RIG-I (1–925). As a negative control, we examined RIG-I containing a mutation in the Walker A box of the helicase ATP binding site (RIG-I-K270A). We find that the RIG-I-ΔRD ATPase is essentially inactive with not only _ppp_ssRNA and _OH_dsRNA, but is inactive as well with poly-G/C, poly-A/U and BTV dsRNA (closed circles, fig 6, and data not shown). Unexpectedly, we found that the RIG-I-ΔRD ATPase was nevertheless clearly stimulated by poly-I/C (albeit not as efficiently as wt RIG-I; fig 6E). In addition, the RIG-I-ΔCARD/ΔRD ATPase was hyper-stimulated by poly-I/C as compared to that of wt RIG-I (fig 6E). These latter results rule out the possibility that the RIG-I ATPase somehow requires the presence of the RD for its activity. These results are thus more consistent with the binding of _ppp_ssRNA and _OH_dsRNA to overlapping sites on RD. It is also possible that the binding of _OH_dsRNA to RD is helped more by its simultaneous interaction with the helicase core than the binding of _ppp_ssRNA to RD. In summary, the ability of poly-I/C to stimulate the ATPase of RIG-I is clearly different from that of heteropolymeric dsRNA. It appears that poly-I/C has the unique ability to bind to both the helicase domain and the RD, and can stimulate the helicase ATPase independently of the RD.

### Stimulation of the RIG-I ATPase is necessary but insufficient for IFNβ activation *in vivo*

**Figure 8:**
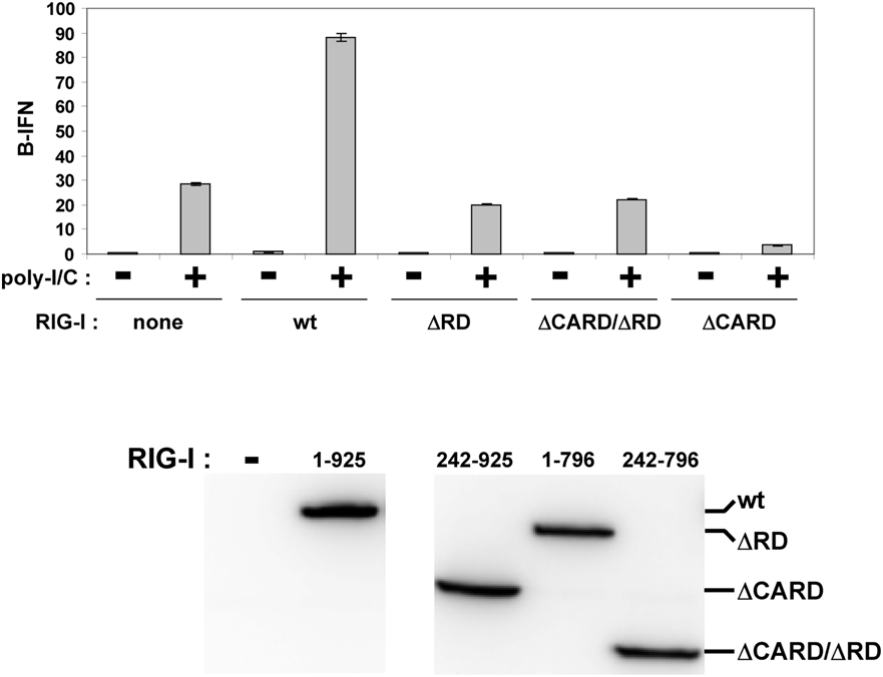
Over-expression of RIG-I variants and poly-I/C induced IFNb activation. Parallel cultures of wt MEFs were transfected with pIFNβ-(ff)luciferase and pTK-(ren)luciferase. Some of the cultures were also transfected with various forms of flag-tagged RIG-I, as indicated below. 24 h later, the cultures were transfected (or not) in duplicate with 5 ug of poly-I/C. Cell extracts were prepared after a further 20 h, and their luciferase activities were determined. Equal amounts of cell extracts (total protein) were Western blotted with anti-flag.

Given that the RIG-I-ΔRD ATPase is stimulated by poly-I/C, we examined whether poly-I/C could activate IFNβ *in vivo* via this variant of RIG-I. When additional wt RIG-I was expressed in wt MEFs, IFNβ activation in response to a fixed amount of poly I/C increased ca. 3-fold (fig 8). However, when additional RIG-I-ΔRD or RIG-I-ΔCARD/ΔRD was expressed, there was little or no effect on poly-I/C induced IFNβ activation. When additional RIG-I-ΔCARD was expressed, this variant acted as a dominant-negative inhibiter of poly-I/C induced IFNβ activation, as expected. Similar results were obtained when _ppp_ssRNA, rather than poly-I/C was used to induce IFNβ activation (data not shown). Thus, stimulation of the ATPase is necessary but not sufficient for poly-I/C induced IFNβ activation. These results are consistent with the finding that the RD is required for RIG-I self-association, and that this self-association is required for downstream signaling [6], [9]. The finding that poly-I/C also competed with the binding of RIG-I to _ppp_RNA (which clearly binds to the RD)(fig 7) suggests that poly-I/C binds to both the helicase core and the RD. These latter results are consistent with the demonstration that the binding of _ppp_RNA or short dsRNA to RIG-I protects a 17kD fragment from trypsin digestion (essentially the entire RD), whereas the binding of poly-I/C produces a 66 kD fragment of the helicase core and the RD [25].

## Discussion

The dsRNAs generated during nondefective SeV infection (due to convergent transcription) or to copyback DI infection (due to intramolecular annealing of the DI genome's complementary ends) would both contain one 5′ tri-phosphate end. As RIG-I is thought to be activated by _ppp_RNA independent of its single- or double-strandedness [7], [8], the finding that IFNβ activation during SeV C^minus^ and DI-H4 infections require RIG-I was not unexpected. dsRNA devoid of free 5′ tri-phosphate ends, however, is normally found only during (e.g., picornavirus) infections that initiate RNA synthesis with protein primers, and these infections activate IFNβ via mda-5 rather than RIG-I. The finding that SeV-GFP+/− infections also requires RIG-I (and not mda-5; figs 1 and 2) was thus not expected. This latter finding, however, is consistent with the recent report that _ppp_ssRNA and short _OH_dsRNA bind to overlapping sites of the RIG-I RD, and that short dsRNAs will activate IFNβ via RIG-I upon transfection if they contain 5′ mono-phosphates that apparently stabilize the dsRNAs intracellularly [25]. We have confirmed that short _OH_dsRNA competes with _ppp_ssRNA for its binding to RIG-I, whereas _OH_ssRNA is inactive (fig 7).

Even though it has been reported that RIG-I by itself does not respond to poly-I/C in mda-5^−/−^ MEFs [12], [13], especially when poly-I/C of 4–8 kbp in length is used [26], this does not exclude the possibility that RIG-I participates in the cellular response to poly-I/C when both helicases are present, or that RIG-I can directly respond to poly-I/C when RIG-I levels are elevated. When RIG-I levels are gradually increased in MEFs, the level of IFNβ activation is proportional to that of RIG-I expression in response to either poly-I/C or _ppp_ssRNA (fig 5). There is other indirect evidence that RIG-I participates in the poly-I/C induced IFNβ activation when both helicases are present. For example, whereas Huh 7 cells respond well to poly-I/C, a sub-line of these cells containing an incapacitating mutation in a CARD of RIG-I (Huh 7.5 cells) has lost most of its response to this dsRNA [27]. In addition, even though the ability of paramyxovirus V proteins (including that of SeV) to bind mda-5 and prevent poly-I/C induced signaling is well documented [28], [29], it is the SeV C protein (that inhibits RIG-I signaling) and not the SeV V protein which acts to inhibit the cellular response to poly-I/C in a SeV infection [15].

We found that both short dsRNAs containing two 5′ OH ends (dsRNA-tr41) and BTV (reovirus) dsRNAs stimulate the RIG-I ATPase *in vitro* almost as efficiently as _ppp_ssRNA (fig 6). Unexpectedly, we also found that poly-I/C stimulated the ATPase of RIG-I variants lacking the C-terminal RD, in strong contrast to either _ppp_ssRNA or heteropolymeric dsRNA (fig 6). Poly-I/C is an unusual dsRNA, composed of complementary homopolymers, and one strand being composed of the rare base inosine. Poly-I/C has been the transfected dsRNA of choice to study IFN induction for many years, not only because it is commercially available, but because it works so well. It has been known for decades that poly-I/C has a special ability to induce IFN [30]. Using DEAE-dextran to transfect various dsRNAs into chick embryo fibroblasts, the efficiency of IFN induction of poly-I/C was found to be orders of magnitude greater than that of poly-G/C or poly-A/U. Moreover, these differences in activity among the various polynucleotides did not appear to reflect differences in their thermal stability, sensitivity to RNase A, the rate or amount of uptake into the cells or in the rate of intracellular breakdown. Colby and Chamberlin (1969) presciently concluded that the high degree of specificity of the induction process was consistent with the existence of a specific intracellular receptor, most probably a protein. One of the possible reasons for the remarkable ability of poly-I/C to induce IFN may be because, unlike other dsRNAs or _ppp_ssRNAs, it can directly bind to both the RIG-I helicase domain as well as to mda-5, stimulating their ATPases and inducing the conformational changes that liberate the CARDs for interaction with IPS-1. At present, there is no clear understanding of the RNA elements of poly-I/C that mimic the PAMPs generated during mouse picornavirus infections, or why poly-I/C alone among various dsRNAs stimulates the RIG-I-ΔRD ATPase. However, it is becoming increasingly clear that poly-I/C is not a simple analog of dsRNA.

While this work was being prepared for publication, Kato et al [26] reported that the length of dsRNA is important for differential recognition by RIG-I and mda.5. Long poly-I/C (4–8 kbp) was found to be a specific ligand for mda-5, whereas relatively short poly-I/C (300 bp) was a specific ligand for RIG-I. We have examined the length of our poly-I/C by agarose gel electrophoresis and found that it was relatively short (200–600 bp relative to DNA markers), and the maximum length of our GFP dsRNA would be 714 bp. Our results are thus consistent with those of Kato et al.
